# Longitudinal multimodal assessment of peripheral nerve involvement in wild-type transthyretin amyloidosis

**DOI:** 10.1007/s00415-026-14066-8

**Published:** 2026-08-25

**Authors:** Vera E. A. Kleinveld, Julia Wanschitz, Anna Hotter, Maria Ungericht, Petra Sanders, Gerhard Pölzl, Alessandra Fanciulli, Wolfgang N. Löscher, Corinne G. C. Horlings

**Affiliations:** 1 Department of Neurology, Medical University of Innsbruck, Innsbruck, Austria; 2 Department of Cardiology, Medical University of Innsbruck, Innsbruck, Austria

**Keywords:** Wild-type transthyretin amyloidosis, Polyneuropathy, Small-fiber neuropathy, Neurofilament, Amyloid

## Abstract

**Introduction:**

Wild-type transthyretin amyloidosis (ATTRwt) is a progressive disease marked by extracellular transthyretin amyloid deposition, predominantly causing cardiomyopathy. Neurological manifestations are known in hereditary ATTR but remain comparatively understudied in ATTRwt.

**Methods:**

Patients with ATTRwt cardiomyopathy were prospectively evaluated at baseline and after 1 year, alongside healthy controls. Neurological assessment included Neuropathy Impairment Score Lower Limb (NIS-LL), nerve conduction studies, quantitative sensory testing (QST), serum neurofilament light chain (sNfL), and intra-epidermal nerve fiber density (IENFD) from skin biopsies. Symptoms and fatigue were assessed using Norfolk-QoL-DN and Chalder Fatigue scale.

**Results:**

Twenty-three patients were included and compared to controls. Pathological IENFD occurred in 47.8% of patients vs. 4.3% of controls (*p*=0.002). In ATTRwt, except cold detection, all QST parameters were abnormal. Sensory–predominant axonal polyneuropathy was present in 82.6%, with higher NIS-LL and symptom burden (*p*<0.001). sNfL levels were similar between groups. Carpal tunnel syndrome was frequent (82.7%). Polyneuropathy with CTS was more common in ATTRwt (*p*=0.025). At follow-up, we observed no clinical or electrophysiological progression, and no newly diagnosed cases of polyneuropathy.

**Conclusions:**

ATTRwt is associated with mixed fiber neuropathy and frequent entrapment neuropathies which should raise suspicion for ATTRwt. We found subtle clinical progression over 13 months.

## Background

Transthyretin amyloidosis (ATTR) is a systemic disorder characterized by the extracellular deposition of misfolded transthyretin (TTR) proteins, leading to a progressive dysfunction of organs and tissues. ATTR is either inherited (ATTRv, variant) or acquired (ATTRwt, wild type), in which the TTR genetic sequence is normal but age-related factors promote protein instability and amyloid formation [1]. In ATTRv, progressive peripheral neuropathy is common, as evidenced by electrophysiological studies and elevated neurofilament light chain (sNfL) as marker of axonal damage [2, 3]. In contrast, ATTRwt manifests predominantly with cardiomyopathy [4]. However, accumulating evidence demonstrates that peripheral neuropathy is substantially more common in ATTRwt than previously recognized, and a prevalence ranging from 43–74% has been described [5–11]. In ATTRwt, polyneuropathy is mild, predominantly sensory, and length dependent, and generally less severe than in ATTRv [6]. Carpal tunnel syndrome and spinal stenosis are described in 70–94% respectively 11–39% of ATTRwt patients, and may precede the diagnosis over years [12]. Additionally, small fiber neuropathy and cardiovascular autonomic nervous system impairment have been described in this population [9, 10, 13]. In ATTRwt, skin biopsy is increasingly recognized as a sensitive tool for detecting early small nerve fiber impairment [10].

Despite increasing recognition of neurological manifestations in ATTRwt, the extent, characteristics, and progression of peripheral nerve involvement remain insufficiently defined, particularly in prospective and longitudinal settings using multimodal assessment. To address this gap, we conducted a prospective cohort study of ATTRwt patients using a comprehensive approach that included clinical neurological assessments, nerve conduction studies, sNfL measurements, patient-reported outcomes, and skin biopsies over a 1-year follow-up period. By integrating detailed neurological, electrophysiological, and neuropathological evaluations, this study aims to provide a more complete characterization of the spectrum and progression of peripheral nerve involvement in ATTRwt.

## Methods

We included adult patients with ATTRwt-cardiomyopathy treated at the Department of Cardiology, Medical University of Innsbruck, Austria. The diagnosis of ATTRwt-cardiomyopathy was established in patients with a Perugini score ≥2 on bone scintigraphy using 99mTc-labeled 3,3 phosphone−1,2-DPD tracers and exclusion of light chain abnormality, or in case of ambiguity (Perugini score < 2), in the presence of amyloid depositions in endomyocardial biopsy, with typing performed by immunohistochemistry or mass spectrography. In all patients, absence of a pathogenic *TTR* mutation was secured through TTR gene sequencing. Exclusion criteria for healthy controls were the presence of any neurological disease and/or the presence of cardiomyopathy.

The study was approved by the ethics committee of the Medical University of Innsbruck (approval number 1418/2021). All patients and participants provided written informed consent. All procedures were performed in accordance with the 1964 Declaration of Helsinki, its later amendments, and the European Data Protection Regulation.

### Clinical characteristics

Symptoms related to cardiac dysfunction were classified according to New York Heart Association (NYHA) functional classification [14]. Comorbidities for polyneuropathy were defined as diabetes mellitus, renal insufficiency (eGFR <60 mL/min/1.73m^2^), hypothyroidism with or without thyroxin substitution, vitamin B abnormalities, chemotherapy, and paraproteinemia.

### Questionnaires

Patients’ subjective perceptions of symptoms associated with nerve fiber damage were assessed using Norfolk Quality of Life-Diabetic Neuropathy (Norfolk-QoL-DN) (range −4–136, higher scores indicate greater impairment) [15]. Fatigue was assessed using the Chalder Fatigue Scale (CFS) (range 0–10, higher scores represent more fatigue) [16].

### Clinical neurological assessments

A detailed neurological history was taken including symptoms associated with carpal tunnel syndrome (CTS), ulnar entrapment neuropathy (UN), and spinal canal stenosis. The Neuropathy Impairment Score Lower Limb (NIS-LL) was used to quantify neurological function (range 0–88 with subscales for muscle weakness (range: 0–64), sensory loss (range: 0–16), and decreased muscle stretch reflexes (range: 0–8). Higher scores indicate greater neuropathic impairment [17].

### Nerve conduction studies

All nerve conduction studies were performed by experienced neurologists using a Dantec Keypoint G3™ EMG system using standard procedures. Both compound muscle action potentials (CMAP) and antidromic sensory nerve action potentials (SNAP) were recorded after supramaximal stimulation, from baseline to first negative peak and peak to peak, respectively. Distal motor latency (DML) and sensory and motor nerve conduction velocities (NCV) were registered. Additionally, F-waves were recorded from the abductor hallucis brevis muscle following tibial nerve stimulation at the ankle.

Normative values as per local protocol were applied. Electrodiagnostic evidence for polyneuropathy was defined as abnormalities in nerve conduction studies involving at least two anatomically distinct peripheral nerves, consistent with a length-dependent polyneuropathy, one of which being the sural nerve [18].

### Quantitative sensory testing (QST)

A QST battery consisting of 10 parameters that represent measures of all relevant modalities of the somatosensory system was used [19]. All components of the QST-battery were performed on the dorsum of the right foot.

Tests for thermal detection were performed using TSA 2 Thermal Sensory Analyzer (Medoc Ltd 2019, Israel). Cold and warm detection thresholds (CDT and WDT), obtained with ramp stimuli of 1 °C/s, were measured first, followed by cold and warm pain thresholds (CPT and WPT). The thresholds of four detection thresholds and three consecutive pain thresholds were averaged. Cutoff temperatures were 0 and 50 °C.

Vibration-detection thresholds (VDT) were determined using a Rydel–Seiffer graded tuning fork (8-point scale, 64 Hz), and age-specific normative values were used [19]. Semmes–Weinstein monofilaments (gradient forces 0.0064–24 g) in descending order were used to determine the mechanical detection threshold (MDT). MDT was defined as the last filament that could be detected by the patient. Mechanical pain sensitivity (MPS) was measured using seven pinprick stimulators with forces between 8 and 512 mN in ascending order. The mechanical pain threshold (MPT) was the first pinprick causing pain. Patients were instructed to quantify the pain experienced after a pinprick on a 0–10 numerical scale, with higher numbers corresponding to more painful stimuli. For determining the wind-up ratio (WUR), ten repetitive pinprick stimuli (256 mN) were applied within the same area with 1 s intervals. Dynamic mechanical allodynia (DMA) was measured using a standardized brush which was applied three times, with a stroke of circa 2 cm over the skin, on a scale of −10 to +10 (ranging not pleasant to pleasant).

### Neurofilament light chain

Serum was collected using CAT Clot activator tubes at room temperature. Neurofilament light chain (NfL) was measured in serum at the commercial laboratory of the University of Ulm (Germany) using standard ELISA techniques, with a limit of detection of 0.37 pg/mL, and evaluated against age-specific reference values derived from healthy controls (*n* = 1033); a Z-score < 1 was considered within the normal range. Serum NfL was measured at follow-up, as this was not yet available at the time of baseline assessment.

### Skin biopsy

3-mm punch skin biopsies were performed under local anesthesia with mepivacaine 1% at the distal (10 cm proximal of the lateral malleolus) and proximal (10 cm distal of trochanter major) leg on the right side. The tissue was immediately immersed in 4% buffered paraformaldehyde for 30 min at room temperature, then washed three times with 0.1 M phosphate-buffered saline (PBS) and transferred to cryoprotective 10% saccharose in 0.1 M phosphate buffer for at least 24 h at 4 °C. Subsequently, biopsies were snap frozen in liquid nitrogen and stored at − 80 °C until use.

The frozen tissue was prepared in 40 µm sections and immunostained for the pan-axonal marker protein gene product 9.5 (PGP 9.5, rabbit polyclonal antibody, dilution 1.400). The antibody was diluted in 1% BSA and 0.3% TritonX and applied to the sections overnight at 4 °C. On the following day, sections were washed three times with PBS and incubated with Alexa Fluor (AF) 488 for 60 min at room temperature (diluted 1:400; Invitrogen) and covered with Vectashield mounting medium containing 4’,6-diamidino-2-phenylindole (DAPI) (H-1200; Vector Laboratories). Using a fluorescence microscope (Leica DFC300 FX), intraepidermal nerve fiber density (IENFD) was calculated using generally agreed upon counting rules [20]. IENFD values were compared to normative values based on a cohort of healthy controls [21], and biopsies were considered abnormal if the IENFD was lower than anticipated for age and gender (< 5th percentile).

### Statistical methods

Data distribution was analyzed using Shapiro–Wilk tests and visually using Q–Q plots and histograms. Patients’ clinical characteristics and demographics were summarized using descriptive statistics. Normally distributed variables are described using mean and standard deviations and non-normally distributed variables are expressed as median and 95% confidence interval. The assessment of intergroup differences was performed using Mann–Whitney U test for non-normally distributed variables and Student’s T-test for normally distributed values. Pearsons’ correlation coefficient was calculated to explore the relationship between categorical and non-categorical variables. Fisher’s exact test and Z-test for proportions were used to compare frequencies. Due to a lack of normative data of quantitative sensory testing in the elderly population, quantitative sensory testing data were transformed using Z-scores obtained from the data obtained in our healthy reference population. Z-scores [Z= (single patient X-healthy control mean)/healthy control SD] were calculated for each QST result. *Z*-Values ≥ 2 were considered abnormal. A constant of 0.1 was added to the rating scales to avoid zero values.

IBM® SPSS® statistical software (27.1.1.0) and GraphPad Prism version 9.4.1 for Windows (Graphpad Software, Boston, Massachusetts USA, www.graphpad.com) were used for statistical analysis. A two-sided p of ≤ 0.05 was set as statistically significant.

## Results

### Part 1. Spectrum of neurological manifestations in ATTRwt

We prospectively included 23 patients with ATTRwt-cardiomyopathy between December 2022 and October 2024. Demographic and clinical data at inclusion are shown in Table 1. In one patient, we found persistent renal insufficiency at follow-up, with a baseline estimated glomerular filtration rate (eGFR) of 38 mL/min. Of the patients with DM, mean Hba1c was 7.4 mmol/mol (range 5.8–8.6). Patients diagnosed with hypothyroidism were all treated and TSH/FT4 values were within normal range. Treatment experienced patients (*n*=17) were treated with a TTR stabilizer (*n*=13), or additionally with a TTR silencer/placebo (*n*=4) as part of a clinical trial.

**Table 1:** Demographical and clinical data of included ATTRwt patients and healthy controls

	ATTRwt (n=23)	Healthy controls (*n*=23)	*p*-value
Age (y)	78.5 (± 4.9)	75.0 (± 4.9)	0.013*
BMI (kg/m^2^)	25.3 (± 3.2)	25.3 (± 3.2)	0.204
Sex, male (*n* (%))	20 (87.0%)	14 (60.9%)	0.044*
Time since diagnosis (m)	10 (CI 8.6–30.6)	n.a	n.a
Comorbidities DM Persistent renal insufficiency Past chemotherapy Treated hypothyroidism Vit B disorders None	6 (26.1%) 1 (4.3%) 0 (0.0%) 8 (34.8%) 0 (0.0%) 10 (43.5%)	0 (0.0%) 0 (0.0%) 0 (0.0%) 0 (0.0%) 0 (0.0%) 23 (100%)	^a^
NYHA No cardiomyopathy 1 2	0 (0.0%) 3 (13.0%) 20 (87.0%)	23 (100%) 0 (0.0%) 0 (0.0%)	< 0.0001*

Demographic data were obtained at the time of inclusion. *BMI* body mass index, *CI* confidence interval, *DM* diabetes mellitus, *m* months, *NYHA* New York Heart Association Classification, *n.a* not applicable, *PND* polyneuropathy disability scale. Values indicated with * are statistically significant (*p* ≤ 0.05). ^a^sample sizes too small to perform statistical analyses

Based on medical history at baseline, 16 (69.6%) patients had a confirmed diagnosis of carpal tunnel syndrome (CTS), and this was bilateral in 12 patients. CTS preceded ATTRwt diagnosis by a median of 9 years (range 2–22 years). Among healthy controls, three (13.0%) participants had a confirmed history of bilateral CTS (compared to ATTRwt, *p* <0.001) and none had a history of unilateral CTS.

Spinal stenosis (L4/L5 in two patients, L3–L5 in one patient) was present in three patients, with diagnoses occurring at the time of ATTRwt diagnosis, 4.5 years before ATTRwt diagnosis, and 13 years before ATTRwt diagnosis. All three patients with spinal stenosis underwent successful surgical treatment and had no clinical symptoms after surgery. Ulnar entrapment neuropathy (UN) occurred in two ATTRwt patients (0 and 11 years prior to ATTRwt diagnosis) and in none of the healthy controls.

### Patient-reported outcomes

At baseline, median Norfolk-QoL-DN score was 16 (8.7–23.7) for ATTRwt patients and 0 (0–4.5) for healthy controls (*U* = 431.5, *p* < 0.001). The Chalder Fatigue scale was not different between groups (*U* = 303.0, *p* = 0.377). Norfolk-QoL-DN correlated with the presence of electrophysiological signs of polyneuropathy in both ATTRwt and healthy controls (*ρ* = 0.461, *p* = 0.001).

### Large nerve fiber function

Table 2 shows the neurological symptoms and results of physical examination. Median NIS-LL was significantly higher in ATTRwt patients compared to healthy controls (8 (IQR 6.6–11.5) versus 0 (IQR 0.4–2.7)*, U* = 454.0,* p* < 0.001). No objective weakness was detected in any of the muscles assessed by the NIS-LL in either the patient or the healthy control group.

**Table 2:** Neurological symptoms and physical examination findings in ATTRwt and controls

	ATTRwt	Healthy controls	*p*-value
*Clinical examination of the lower leg*			
Reduced or absent ankle reflexes	18 (78.2%)	9 (39.1%)	0.007*
Reduced or absent knee reflexes	7 (30.4%)	3 (13.0%)	0.153
Vibration impaired or absent	17 (73.9%)	4 (17.4%)	<0.01*
Proprioception impaired or absent	5 (21.7%)	0 (0.0%)	^a^
Allodynia	1 (4.3%)	0 (0.0%)	^a^
Muscle weakness	0 (0.0%)	0 (0.0%)	^a^
*Symptoms in the lower leg*			
Tingling, pins and needles	11 (47.8%)	5 (21.7%)	0.120
Electric pain	2 (8.7%)	1 (4.3%)	1.00
Numbness	13 (56.2%)	5 (21.7%)	0.033*
Pain	3 (13.0%)	1 (4.3%)	0.608
Other unusual sensations	4 (17.4%)	0 (0.0%)	0.109
Subjective weakness	8 (34.8%)	1 (4.3%)	0.022*

*ATTRwt* wild-type transthyretin amyloidosis. Values indicated with * are statistically significant. ^a^sample size too small to perform statistical analysis

**Fig. 1:**
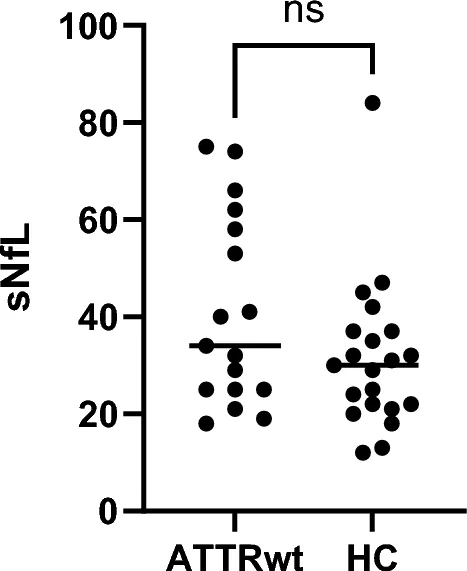
Serum neurofilament light chain in ATTRwt and healthy controls, in pg/mL. *ATTRwt*: wild-type transthyretin amyloidosis, *HC* healthy controls, *sNfl* serum neurofilament light chain, *ns* not significant

sNfL was available in 17 ATTRwt patients and 21 healthy controls and was similar in both groups: median 34.00 (IQR 30.88–51.12) vs median 29.5 (IQR 24.21–38.70) pg/mL,* U* = 2,258, *p* = 0.133, Fig. 1). sNfL was not different in treatment-naïve patients compared to patients receiving disease-modifying treatment (*p* = 0.733).

Nineteen patients (82.6%) met the electrodiagnostic criteria of a mixed sensory/motor large fiber polyneuropathy. Among these, 18 patients exhibited a pure axonal damage pattern, while one patient also exhibited additional demyelinating features. Ten healthy controls (43.5%) met the electrodiagnostic criteria of an axonal large fiber mixed sensory/motor neuropathy (frequency of polyneuropathy compared to ATTRwt patients, *p* = 0.0134).

In the ATTRwt group, pathological sural SNAPs were accompanied by pathological peroneal CMAPs in 14 patients, pathological tibial CMAPs in 12 patients, and pathological superficial radial nerve SNAPs in 5 patients.

Among healthy controls, pathological sural SNAPs were accompanied by pathological peroneal CMAPs in five individuals, pathological tibial CMAPs in six individuals, and pathological superficial radial nerve SNAPs in two individuals.

**Fig. 2:**
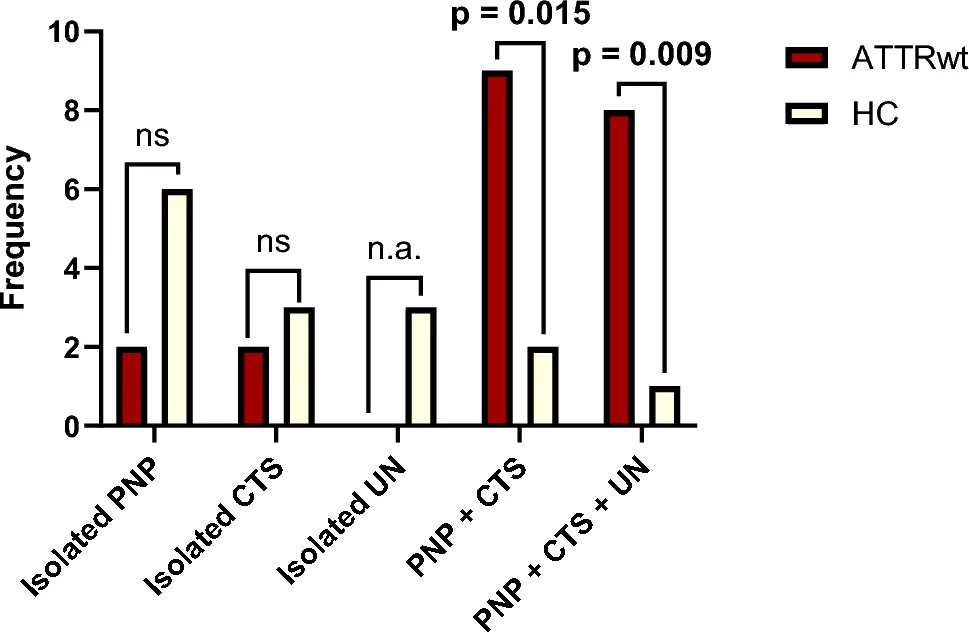
Distribution of peripheral nerve abnormalities on nerve conduction studies among ATTRwt patients and controls. *HC* healthy controls, *ATTRwt* wild-type transthyretin amyloidosis, *CTS* carpal tunnel syndrome, *UN* ulnar entrapment neuropathy, *PNP* polyneuropathy. Values indicated in bold are statistically significant

Electrophysiological CTS was more common in the ATTRwt population (19/23, 82.6%) than in healthy controls (7/23, 30.4%, *p* < 0.01). In ATTRwt patients with CTS, it was bilateral in 84.2%, and in healthy controls, in 57.1%. Electrophysiological UN at the elbow was seen in eight (34.5%) ATTRwt patients and four (17.4%) healthy controls (*p* = 0.179). UN was bilateral in 37.8% of ATTRwt patients with UN and in none of the healthy controls, Table 3. The combination of polyneuropathy+CTS and polyneuropathy+CTS+UN was more common in ATTRwt than in healthy controls (Fig. 2). We did not identify any patients or controls with the combination of polyneuropathy+UN without CTS (Fig. 2).

**Table 3:** Nerve conduction studies in patients and healthy controls fulfilling the criteria of polyneuropathy

		Ref. value	ATTRwt (n=19)	Healthy controls (n=10)	P-value
Peroneal nerve	CMAP (mV) dML (ms)	>2 <6.0	1.18 (0.75–1.95) 5.24 (3.33–5.47)	3.15 (2.06–3.00) 4.56 (4.11–5.04)	**0.003** 0.356
Tibial nerve	CMAP (mV) dML (ms) FWL (ms)	>5 <6.5 *	1.48 (1.06–3.14) 4.74 (4.12–5.69) 63.3 (51.31–68.10)	4.55 (3.17–6.27) 4.07 (3.58–4.49) 55.1 (53.10–58.78)	**<0.001** **0.009** **0.004**
Sural nerve	SNAP (µV) NCV (m/s)	>10 >40	1.89 (1.21–6.72) 33.00 (13.33–33.57)	9.05 (6.10–12.88) 44.50 (40.95–47–77)	**0.006** **<0.001**
Superficial radial nerve	SNAP (µV) NCV (m/s)	>16 >55	22.75 (15.43–28.54) 54.25 (45.63–59.11)	20.10 (14.02–18.71) 55.35 (51.85–59.65)	0.464 0.588

Data are presented as median with 95% confidence interval. *ATTRwt* wild-type transthyretin amyloidosis, *SNAP* sensory nerve action potential, *CMAP* compound muscle action potential, *NCV* nerve conduction velocity, *DML* distal motor latency, *FWL* F-wave latency, *µV* microvolts, *mV* millivolts, *ms* milliseconds, *m/s* meters per second. *height-dependent normative values

### Small nerve fiber function

Table 4 shows the results of the small fibre assessments using QST and skin biopsy. At baseline, 20 ATTRwt patients and all healthy controls consented for skin biopsy. Eleven (47.8%) ATTRwt patients had a reduced distal IENFD according to age- and sex-specified normative values, all exhibiting a pathological distal to proximal IENFD ratio (< 0.9) suggesting a length dependency. Only one healthy control showed a reduced IENFD (*p* = 0.002). Median distal IENFD was 2.78 (± 2.04) in ATTRwt and 7.77 (± 3.16) in HC (IQR 4.7/mm), *p* < 0.001. All but one patient with a reduced IENFD (90.9%) had electrophysiologically confirmed polyneuropathy.

**Table 4:** Small nerve fiber assessment by quantitative sensory testing and skin biopsy in patients with ATTRwt and healthy controls

	ATTRwt	Healthy controls	*p*-value
*Thermal QST*			
CDT (°C)	20.2 (12.9–21.4)	18.4 (13.9–22.2)	0.886
WDT (°C)	44.7 (41.0–45.1)	44.6 (42.5–45.9)	0.684
CPT (°C)	2.4 (3.6–11.2)	0.01 (0.5–3.6)	0.055
HPT (°C)	49.7 (41.1–50.0)	48.7 (47.3–49.3)	0.775
*Tactile QST*			
MDT (grams)	1.7	0.064	0.051
MPT (mN)	64	16	0.156
MPS (0–10 scale)	3	1	0.221
DMA (0–10 scale)	5	6	0.073
WUR	1	1	0.213
VDT	4	6.7	0.030*
*Skin biopsy*			
Proximal IENFD*	7.55 (± 2.78)	11.7 (± 3.49)	<0.001*
Distal IENFD*	2.78 (± 2.04)	7.77 (± 3.16)	<0.001*
IENFD ratio*	0.37 (± 0.06)	0.80 (± 0.49)	<0.001*

^*^20 of 23 ATTRwt patients and all healthy controls consented for skin biopsy

*CDT* cold detection threshold, *WDT* warm detection threshold, *CPT* cold pain threshold, *HPT* heat pain threshold, *MDT* mechanical detection threshold, *MPT* mechanical pain threshold, *MPS* Mechanical pain sensitivity, *DMA* dynamical mechanical allodynia, *IENFD* intraepidermal nerve fiber density, *WUR* wind-up ratio, *VDT* vibration detection threshold. Values indicated with * are statistically significant

Based on our healthy population, for QST parameters, despite the absence of a significant difference at the group level (Table 3), we identified multiple patients with abnormal Z scores for QST parameters. In thermal QST, abnormal Z-scores were seen for CDT in zero (0%) patients, for WDT in four (17.4%) patients, for CPT in ten (43.5%) patients, and for HPT in five (21.7%) patients.

In tactile QST, abnormal Z-scores were seen for MDT in seven (30.4%) patients, for MPT in five (21.7%) patients, for MPS in 14 (60.9%) patients, for DMA in 14 (60.9%) patients, for WUR in eight (34.8%) patients, and for VDT in five (21.7%) patients. A statistically significant moderate correlation was only found between vibration sense and the IENFD ratio (*r* = 0.465, *p* = 0.039), but for none of the other QST measures.

### Part 2. Progression of neurological complications

A follow-up visit was performed 12–16 months after the baseline visit, with a median follow-up duration of 13 months. Four patients were lost to follow-up (*n*=3 no longer willing to participate, *n*=1 deceased). During follow-up, TTR-specific therapy remained unchanged.

For patients with paired data, the NIS-LL score was significantly higher at follow-up compared to baseline (9.9±5.5 versus 11.4±6.2, p < 0.001). At follow-up, similar to the assessment at baseline, no motor involvement was seen in any of the patients. Symptom burden, as assessed by Norfolk-QoL-DN, remained unchanged (14 (5.8–23.2) versus 10 (9.2–30.5), *p* = 0.078). Relevant progression of polyneuropathy in nerve conduction studies was absent in all patients. No additional patients with polyneuropathy, CTS or UN could be identified. For CTS, one patient improved and one patient progressed. For the patients with ulnar entrapment neuropathy, one patient improved and one patient with bilateral ulnar entrapment showed unilateral progression. All patients with any form of progression as assessed by nerve conduction studies during follow-up received TTR-specific treatment.

For small nerve fiber parameters, QST thermal parameters remained stable comparing baseline and follow-up data (CDT *p* = 0.590, WDT *p* = 0.979, CPT *p* = 1.00 and HPT *p* = 0.247). For tactile parameters, only vibration sense significantly worsened (median 4 IQR 2.7–5.0 at baseline vs median 0 IQR 0.7–3.4 at follow-up, *p* = 0.039).

**Fig. 3:**
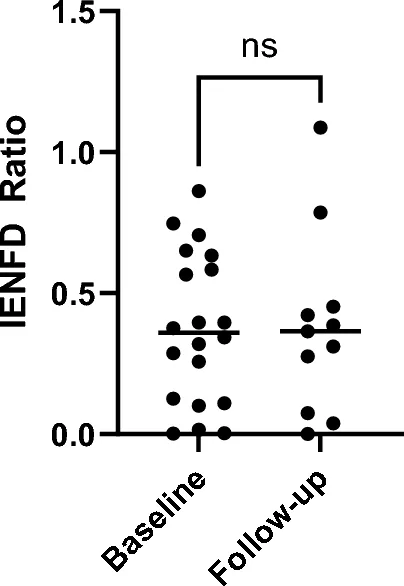
Intraepidermal nerve fiber density ratio in ATTRwt at baseline and follow-up. *IENFD* intraepidermal nerve fiber density, *ns* not significant

At follow-up, median IENFD at the distal leg was 1.6 (IQR 0.57–5.32) and the median distal to proximal IEFND ratio was 0.36 (IQR 0.16–0.60), both remained unchanged compared to baseline (*p* = 0.670 and* p* = 0.919, respectively). On an individual level, three additional patients (33.3% of patients with normal IENFD at baseline) fulfilled the criteria for abnormal distal IENFD at follow-up (see Fig. 3).

At follow-up, fatigue scores remained unchanged in the ATTRwt cohort (*p* = 0.884).

## Discussion

This prospective study of a cohort of ATTRwt patients evaluated the presence of neurological large-fiber and small-fiber impairment and its progression over time. Our comprehensive set of clinical investigations revealed peripheral nerve involvement in the majority of ATTRwt patients. As ATTRwt is a disease manifesting exclusively in the elderly, age-related comorbidities for polyneuropathy and the sole effect of aging on peripheral nerve function affect clinical parameters and nerve conduction studies [22]. However, we were able to demonstrate that the proportion of patients with peripheral nerve complications in our ATTRwt cohort was significantly higher than in age-matched controls.

Previous studies showed evidence of amyloid deposits in nerve biopsies in ATTRwt patients [23] as was discovered before in ATTRv [24], suggesting a possible direct pathogenic effect in addition to indirect toxic effects of TTR on nerves [24]. We found electrophysiological signs of a mild to moderate axonal sensory predominant polyneuropathy in 82.6% patients, which is consistent with previous studies [12, 23, 25]. The relatively high prevalence (43.5%) of electrophysiological abnormalities in our healthy control cohort compared with previous population-based estimates of polyneuropathy [26, 27] is likely due to the lack of age-specific normative values in our study. However, as identical reference values were applied to both the ATTRwt and healthy control groups, this limitation is unlikely to have biased the between-group comparison, and the substantially higher prevalence of polyneuropathy in the ATTRwt group remains noteworthy.

In line with this, the majority of patients reported symptoms associated with polyneuropathy. Clinical investigations revealed predominantly sensory signs and symptoms with preserved motor function, which was also reflected in nerve conduction studies. Also in previous cohorts, no relevant motor impairment has been described [6, 23]. At follow-up, no patients showed electrophysiological progression of polyneuropathy. NIS-LL scores increased at follow-up with an observed change of 1.7 points. Generally, a 2-point change in NIS-LL represents a clinically meaningful difference [28], and this benchmark also used in controlled trials on transthyretin-specific therapy to identify treatment responders [29]. Other trials on transthyretin-specific therapies used an even higher threshold of 4.7 points change to define treatment responders [30].

The observed increase in our cohort did not reach these predefined criteria for clinical relevance. Nevertheless, the increase in NIS-LL may reflect subtle disease progression that was not captured by electrophysiological measures. In addition, nerve conduction studies are subject to inter-rater and inter-laboratory variability, which may further limit their sensitivity for detecting small longitudinal changes. Given the relatively short median follow-up duration of 13 months and the slowly progressive nature of ATTRwt-associated neuropathy, our findings support the absence of rapid progression rather than the absence of progression altogether. Longer longitudinal studies are needed to better characterize the rate and extent of neurological progression in this population.

In line with previous studies [23, 25, 31, 32], we found CTS in a large proportion of patients (82.6%), which was bilateral in 84.2% of patients with CTS, and was more common in ATTRwt patients than in healthy controls. Bilateral CTS can be considered a significant red flag for ATTR [33]. Also, the coexistence of polyneuropathy and CTS may constitute another important clinical red flag, as warranting further diagnostic evaluation for ATTRwt. Although the process of extracellular amyloid accumulation in the connective tissues [34] could theoretically affect the ulnar nerve at common entrapment sites, we did not find evidence of prevalent ulnar neuropathy in our cohort.

It was previously described that NfL in serum is a sensitive marker of axonal damage and is elevated in ATTRv, whereas it is only elevated in ca. 10% of ATTRwt patients [25]. In a cohort of ATTRwt patients with severe neuropathy, NfL was markedly raised [7]. In our ATTRwt cohort, using a conventional ELISA assay, NfL did not demonstrate difference between the groups. As ATTRwt patients with severe polyneuropathy were not present in our cohort, this supports the idea that NfL levels reflect severity of peripheral nerve affection [25]. ELISA was used to measure sNfL as this was the commercially available assay at the time the study was conducted. Previous studies have demonstrated that conventional ELISA assays yield NfL concentrations within a comparable range to ultrasensitive methods in clinical populations [35]. Larger prospective studies incorporating highly sensitive assays and serial NfL measurements are needed to better define NfL dynamics and their relationship to ATTRwt progression.

We found a reduced IENFD in most patients, which is similar to the reduction of IENFD in ATTRv [36]. IENFD reduction followed a proximal to distal gradient, which suggests a length depending process, as in large fiber neuropathy in ATTRwt. Even though the small-diameter nerve fibers evaluated in the skin biopsy are responsible for nociception and temperature sensation, IENFD did not correlate to the majority of QST elements, as is also described by others [37, 38]. We argue that IENFD measurement only provides a quantification of nerve fibers, but not an assessment of the functionality of the remaining nerve fibers [39]. Also, nerve fiber density can vary significantly between different skin regions, whereas QST results might reflect more generalized sensory processing. IENFD showed no relevant change over time, likely reflecting a floor effect due to already markedly reduced IENFD at baseline, limiting the potential for further measurable decline. Most patients (90%) exhibited a mixed large- and small-fiber neuropathy, whereas isolated small-fiber neuropathy (indicated by reduced IENFD) was only seen in one patient.

### Strengths and limitations

The main limitation of this study is its relatively small sample size, due to the rareness of ATTRwt. We reported time since diagnosis, which does not necessarily reflect true disease duration, as diagnostic delay is common in ATTRwt. Determining the interval from first symptom onset was not feasible, since early manifestations of ATTRwt cardiomyopathy are often subtle and nonspecific. Relevant comorbidities that may contribute to peripheral neuropathy or complicate the interpretation of nerve conduction studies were present in a subset of patients, which may have limited causal attribution of electrophysiological abnormalities to ATTRwt alone. Among the potential alternative causes of peripheral neuropathy, diabetes mellitus was the most relevant comorbidity. Nevertheless, after excluding patients with diabetes, electrophysiological abnormalities remained present in 13 of 17 patients (76.5%), suggesting that diabetes alone does not account for the observed findings. A small proportion of patients had lumbar spinal stenosis; however, this is unlikely to have influenced the estimated prevalence of polyneuropathy, as an abnormal sural SNAP was a key diagnostic criterion and sensory nerve action potentials are typically preserved in lesions proximal to the dorsal root ganglion. As needle electromyography was not performed, it could not be excluded that motor nerve abnormalities in patients with lumbar stenosis were due to a radicular lesion.

In conclusion, the majority of patients with ATTRwt demonstrated electrophysiological evidence of large-fiber neuropathy, most commonly in the form of a mild sensory predominant axonal polyneuropathy, in absence of clinically overt muscle weakness. Overall, symptom burden was low and rapid neurological progression was uncommon. Such a phenotype is frequently classified as chronic idiopathic axonal polyneuropathy (CIAP). From a clinical perspective, ATTRwt should be considered in the differential diagnosis of older patients with unexplained polyneuropathy. An important clinical red flag is bilateral CTS, which was highly prevalent and preceding the diagnosis of ATTRwt by many years.

## Data Availability

Data is available from the author upon reasonable request.
